# Cross-sectional survey exploring current intake practices for dogs admitted to animal shelters in Texas: a descriptive study

**DOI:** 10.3389/fvets.2023.1296425

**Published:** 2023-12-19

**Authors:** Mackenzie Cranford, Abbey Bing, Alissa Cisneros, Amber D. Carroll, Hannah Porter, Anastasia Chiara Stellato

**Affiliations:** Department of Animal and Food Sciences, Texas Tech University, Lubbock, TX, United States

**Keywords:** animal shelter, intake examination, dog, behavior, welfare

## Abstract

**Introduction:**

Entering an animal shelter is a stressful experience for dogs that can impair their welfare, adoptability, and shelter staff safety; thus, it is crucial to reduce the stress experienced during intake. This study investigated the current intake practices for dogs admitted in animal shelters in Texas, United States.

**Methods:**

To gather data, an online survey was designed and distributed to shelter employees responsible for intake at animal shelters. The survey collected information about examination procedures, the type of information collected from owner-surrenders, as well as the housing environment for the dogs.

**Results:**

Survey participants (*n* = 64) were shelter staff from municipal (59%, 38/64) and private shelters (23%, 15/64) in 47 counties. Handling techniques reported to be used during intake exams varied depending on the dog’s behavior, with participants reporting higher restraint for aggressive dogs and lower restraint for calm dogs. If the dog was displaying fear, participants reported offering food and attention (89%, 47/53), using towel restraint (64%, 34/53) and conducting the exam on someone’s lap (49%, 26/53). In cases of aggression, it was commonly reported to use muzzles (81%, 42/52) and catch poles (77%, 40/52), and shorten the exam (71%, 37/52). After the exam, most reported placing dogs on the adoption floor (45%, 27/60) or placing them wherever space was available (20%, 12/60).

**Discussion:**

Results provide descriptive information on current intake procedures and routine handling techniques used in Texas shelters. Future research should explore shelter dog responses to routine handling techniques to support the development of evidence-based protocols during routine intake examinations and procedures.

## 1 Introduction

Reports estimate that about 3.1 million dogs enter shelters every year in the United States (1), with the state of Texas having the second largest intake numbers at approximately half a million in 2023 (2). Entering the shelter environment is highly stressful (3, 4), and one of the first exposures at the facility is the intake examination. These practices are essential to ensure the health and welfare of animals entering the shelter population (5); however, previous research has demonstrated that dogs display fear and aggression during veterinary care (6–9). There are many factors that can contribute to fear and aggression during examinations. These include exposure to novel environments (5, 10) and people (11), separation from owner or familiar caregiver (9), unfamiliar handling, and previous negative handling experiences resulting in pain and/or stress (6). Intake exams in animal shelters could pose similar risks of increased fear and/or stress to shelter animals. Veterinary-related fear is a serious concern as it directly impairs shelter animal welfare and can increase the risk of injury (e.g., bites, scratches) for the staff performing the exam (12). This stress can negatively influence the health and welfare of the shelter population as stress responses can lead to inaccurate diagnostic tests (13), prevent the completion of thorough examinations (14), negatively influence the health and recovery of sick and injured animals (15), and lead to future negative interactions with shelter staff (6).

Strategies for reducing veterinary-related fear include conducting the exam on the ground on a non-slip surface, allowing acclimation to the exam room prior to interactions, provisioning of treats (16–20), using calming pheromones or scents (21, 22), and using minimally invasive restraint methods (20). These methods are suggested to reduce dog stress (23, 24), reduce injury to the handler (16, 25, 26), and create a positive experience for the dog and the staff. Thus, if applied in a shelter environment, these recommendations may support the welfare and rehoming of shelters dogs.

If one of the dogs first experiences with the shelter environment and staff involves a distressing intake examination, this can have detrimental effects on their behavior and health which can subsequently increase their length of stay and impact re-homing success (27, 28). According to the Guidelines for Standards of Care in Animal Shelters (5), shelters must make every effort to minimize stress during intake to prevent the development of behavior problems that may prevent adjustment to the shelter environment and adoption success (29). However, it has yet to be explored what practices and procedures are used by shelters during the intake process. Therefore, we aimed to explore current intake procedures within Texas animal shelters to gain insight into potential ways dog stress can be minimized during intake to a shelter environment.

## 2 Methods

This study was approved by the Institutional Review Boards (IRB# 2021–844) at Texas Tech University in Lubbock, Texas, United States.

### 2.1 Data collection

An online survey was created via Qualtrics^®^. To be eligible to participate, individuals were required to be 18 years of age or older and responsible for intake at an animal shelter in Texas, United States. The survey was distributed using both convenience and virtual snowball sampling to animal shelters across Texas. Virtual snowball sampling involves asking previous participants to share the survey with relevant eligible groups. This form of sampling has been effective in increasing participation within hard-to-reach populations (30). Contact information was collected from animal shelters in each Texas county. Animal shelter staff were asked to complete our survey through email, online contact forms, and direct messaging on social media. To reach participants that may not have otherwise been captured, we used a referral-based method of recruitment by promoting our survey on social media with sharing encouraged. Individual participants were prohibited from completing the survey more than once; however, to capture the full breadth of intake procedures that may occur within an individual shelter, multiple intake personnel from the same shelter were able to complete the survey. To maintain confidentiality and mitigate social desirability bias, we refrained from requesting specific shelter identification other than identifying the county where the shelter is located; consequently, we cannot definitively ascertain the total number of shelters represented in our survey responses. Further, participants had the option to skip questions that they were unsure about or did not want to answer. To encourage participation, respondents were given the opportunity to enter in a lottery for the chance to win a $200 gift card to be used on supplies for the shelter.

### 2.2 Questionnaire

The survey consisted of 42 questions and was divided into 3 sections: (1) intake procedures and information collected (e.g., behavior and health information collected during owner surrenders), (2) intake exam (e.g., personnel, exam environment, handling techniques and tools used), and (3) housing environment (e.g., location of intake exam, housing post-intake). For the full list of questions provided in the survey, see Supplementary materials S1.

No statistical analysis was performed as the primary goal of this survey was to capture a snapshot of current intake procedures and the prevalence of the use of commonly recommended stress-reducing practices. Prevalence, indicating the frequency of specific practices used during intake, was assessed through the calculation of descriptive statistics, and reported using graphical summaries.

## 3 Results

A total of 298 shelters from 141 counties in Texas were contacted. Of those contacted, 64 responses were received from shelter staff employees in 47 counties, thereby representing approximately 19% of Texas. Of the respondents, 59% (38/64) were from municipal shelters, 23% (15/64) were from private shelters, and 17% (11/64) indicated that they work for another type of shelter. Though a total of 64 responses were collected the number of responses varied among the questions.

For managing intakes that are owner surrenders, 72% (46/64) of participants reported always scheduling surrenders except for emergencies, 27% (17/64) reported never scheduling surrenders, and 2% (1/64) reporting being unsure how the shelter manages surrenders. During owner surrenders, most participants reported asking owners for specific behavioral tendencies, such as dog-directed aggression (90%, 52/58), with a minority collecting medical information, such as skin conditions (42%, 24/57; Table 1). A full description of information collected during owner surrender intake is found in Table 1.

**Table 1:** Descriptive statistics of the general (*n* = 63), household (*n* = 62), behavior (*n* = 58), and medical (*n* = 57) information collected from the owner upon surrender.

Information collected	Frequency (Percentage)
General	
Dog age	61 (97)
Dog breed	60 (95)
Dog sex	62 (98)
Duration of ownership	46 (73)
Reason for surrender	62 (98)
Bite history	56 (89)
Number of daily walks	8 (13)
Time left alone	17 (27)
Housetrained	51 (81)
Crate trained	43 (68)
Type of food	26 (41)
Household	
Number of adults	16 (26)
Number of children	22 (35)
Behavior toward people	53 (85)
Behavior toward children	56 (90)
Number of other pets	37 (60)
Type of other animals	46 (74)
Behavior toward other animals	55 (89)
Type of dwelling	15 (24)
Where dog sleeps	26 (42)
When dog stays when alone	27 (44)
Where dog primarily lives	33 (53)
Behavioral	
Fearful tendencies	45 (78)
Stranger-directed aggression	44 (76)
Owner-directed aggression	45 (78)
Dog-directed aggression	52 (90)
Resource guarding	43 (74)
Separation anxiety	36 (62)
Handling sensitivity	24 (41)
House-soiling issues	33 (57)
Excessive vocalization	19 (33)
Destructiveness	34 (59)
Noise-phobic	20 (34)
Chasing behavior	24 (41)
Escape artist	39 (67)
No information collected from owner	2 (3)
Medical	
Gastrointestinal issues	22 (39)
Musculoskeletal issues	19 (33)
Skin conditions	24 (42)
Metabolic	16 (28)
Respiratory issues	19 (33)
Cardiovascular issues	20 (35)
Neurological issues	17 (30)
Acute pain	19 (33)
Chronic pain	19 (33)
Vision	22 (39)
Hearing	23 (40)
No information collected from owner	16 (28)

Regarding the personnel who conduct the intake exam, 36% (20/56) of participants reported the personnel to be animal control officers, 30% (17/56) were animal care workers, 14% (8/56) were veterinary technicians, 5% (3/56) were veterinarians, and 14% (8/56) had other job titles. The majority of participants (54%, 32/59) reported having 2–3 personnel present during the intake exam, and none reported more than 3 personnel. When asked if personnel responsible for intake are required to complete continued education related to dog behavior and welfare, 61% (37/61) reported yes, 28% (17/61) reported no, 10% (6/61) are not encouraged or have no opportunity, and one participant declared to be unsure if this was a requirement.

**Figure 1:**
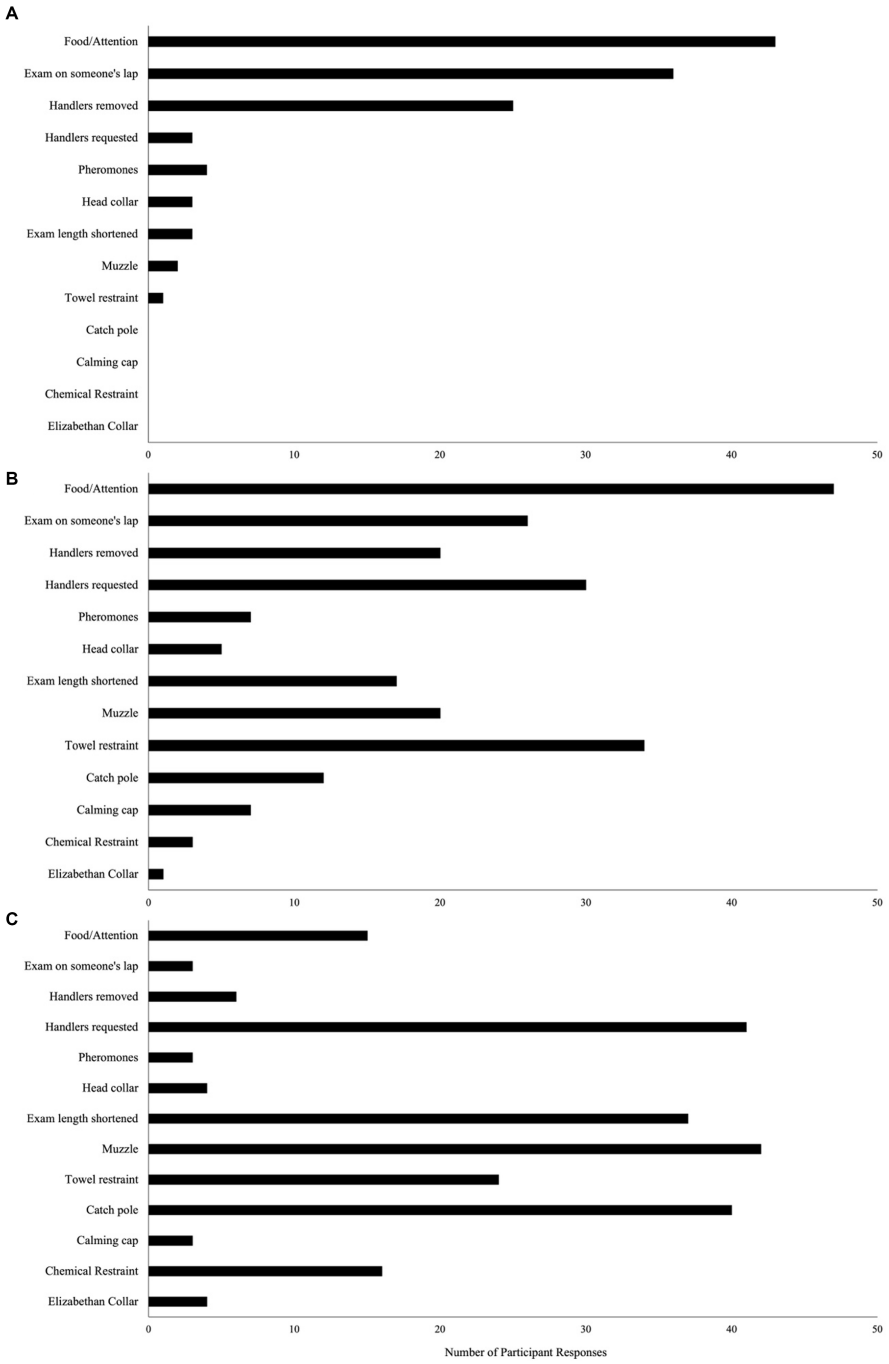
Participants (*n* = 50) reporting the handling techniques used during intake exam, depending upon dog behavior; classified as Calm **(A)**: relaxed, no signs of aggression or fear-related behaviors; Fear **(B)**: lowered posture, ears back, tail tucked, whimpering or whining, shaking/trembling, attempts to hide or escape; Aggression **(C)**: fear-related behaviors plus baring teeth, attempting to bite, growling, lunging.

Throughout the exam, 64% (34/53) of participants reported the dog is approached indirectly (crouching/kneeling on the ground) and 49% (26/53) reported the dog is approached directly (standing/walking directly toward the dog). Before the examination begins, 57% (30/53) of participants reported giving the dog time to explore the exam room, and during the exam 76% (41/54) reported giving the dog lots of attention (e.g., treats, petting, soothing voice). Regarding the handling techniques used during the intake exam, a majority of participants reported using low stress techniques (e.g., food, positive attention); however, as the dog’s demeanor changed from calm to aggressive, there was an increase in reports of using more restrictive methods (e.g., muzzle, catch pole) and of not completing a full examination (Figure 1). For full descriptions of handling techniques, see Supplementary materials S1.

In regard to the location of the examination, 41% (24/59) reported that they never conduct the exam outside; however, 51% (30/59) indicated sometimes and 8% (5/59) indicated always. For large dogs specifically, it was found to be most common to conduct the exam with the dog untethered on the ground (53%, 29/55) and on the ground with a leash tethered to a wall (45%, 25/55). For small dogs, 39% (21/54) reported that they conduct exams on a table without traction surface, 28% (15/54) reported using a traction surface (e.g., non-slip mat), 19% (10/54) reported conducting it on the ground, untethered, and 15% (8/54) reported tethering them to a wall.

**Figure 2:**
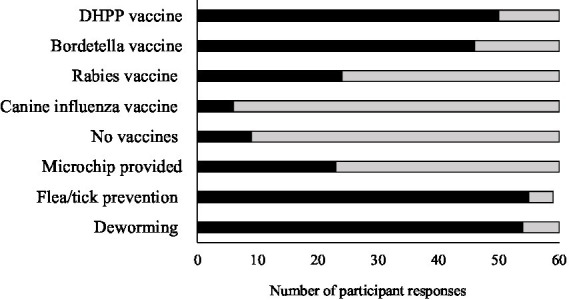
Displaying the vaccines and treatments administered during intake examination based on the following participant responses: Yes (black bars) and No (gray bars).

During the intake exam, participants reported detecting for: skin conditions (86%, 51/59), respiratory issues (59%, 35/59), musculoskeletal issues (54%, 32.59), neurological issues (51%, 30/59), intestinal issues (37%, 22/59), cardiovascular issues (14%, 8/59), and if the animal was underweight (78%, 46/59). They also reported testing for parvovirus (47%, 28/59), heartworms (36%, 21/59), and fever (24%, 14/59). A small portion (10%, 6/59) of participants reported not testing for any medical conditions. Most participants reported providing vaccines (e.g., DHPP (distemper, hepatitis, parainfluenza, parvo), bordetella, rabies and canine influenza), with 15% (9/60) reporting that they do not vaccinate (Figure 2). Shelters reported administering flea/tick control (56%, 33/59) and deworming dogs (72%, 43/60), but a majority did not microchip dogs during intake exams (62%, 37/60).

Upon completion of the intake exam, 45% (27/60) of participants reported housing dogs in a kennel on the adoption floor, 20% (12/60) reported placing dogs wherever space is available, 7% (4/60) reported housing dogs isolated in a closed room, 3% (2/60) reported housing dogs in a kennel within the intake room, and 25% (15/60) of participants reported placing dogs in other rooms following intake.

## 4 Discussion

According to the results of our online survey, a majority of the participants reported using techniques that align with low stress recommendations to mitigate dog stress during veterinary examinations (e.g., provision of treats, acclimation to examination room, indirectly approaching) (20, 31). As shelter dogs require regular handling and medical care throughout their shelter stay, it is imperative that these strategies are used to prevent context learning and create positive veterinary experiences to support the development of positive future interactions.

Results suggest that when shelter dogs are calm, staff opt to use low-stress handling techniques (e.g., treats, pheromones, performing exam on someone’s lap, fewer handlers) with the level of restraint increasing as the dog’s behavior changes from calm to aggressive (e.g., muzzles, catch pole, chemical restraint, adding handlers, and shortening the length of the exam). Similar findings were detected in a previous study where owners with veterinary experience were more likely to agree with using higher restraint on an aggressive dog during routine veterinary exams (32). Recommendations suggest using dog behavior to guide decisions on the appropriate handling used during veterinary exams, with more restrictive techniques applied after first attempting to use a less restrictive technique (20). Due to perceived risk to handler safety, handlers may apply more restrictive handling methods (e.g., muzzle, catch pole) during routine veterinary exams; however, the use of higher restraint on an aggressive dog contradicts the low stress handling philosophy, as it is more likely to increase fear and aggression and thus risk to handler safety (25). In a shelter environment, dog aggression poses a risk to human safety and has major welfare implications, as they are more likely to be returned to the shelter (33) and euthanized (34). Thus, every effort should be made to mitigate stress and thereby prevent the development of aggression within a shelter setting (35). To support the implementation of these practices, future research is needed to assess the influence of commonly used handling techniques on shelter dog behavior and welfare.

Regarding the personnel responsible for conducting the exam, majority reported animal control officers or animal care workers, and a minority reported veterinarians or veterinary technicians. Animal control officers are required to complete annual continuing education and training (36); however, study results reveal that not all personnel responsible for intake are required, encouraged, or given the opportunity to complete continued education related to animal behavior and welfare. Investing in shelter staff training can provide employees with a skillset in using handling that supports dog welfare and enhances their safety around fearful or aggressive dogs (31). It is therefore crucial that continued education on behavior and welfare is encouraged and accessible for all animal shelter staff so they can remain up to date on best practices and improve their handling and interactions with shelter dogs.

After completion of the exam, majority of participants reported housing dogs in a kennel on the adoption floor. The shelter environment is loud, unpredictable, and overstimulating and can lead to the development of acute and chronic stress (37, 38) which can impair adoption success (27). The first week of their stay at the shelter has been suggested to elicit the highest levels of stress (4, 39); thus, it is encouraged to avoid unpleasant experiences until they are adjusted to the environment (31). When entering any novel or potentially stressful environment, it is recommended to allow time to settle and adjust to their new surroundings., Previous research suggests that if a dog acclimates to the environment, they are less fearful, more behaviorally diverse, and more interactive with people and other animals (40, 41). Further research is recommended to explore the influence of providing dogs time to acclimate to the shelter environment before proceeding to standard housing.

Potential barriers to the implementation of low stress strategies in a shelter setting include a lack of evidence-based recommendations and varying levels of resources (e.g., time, space, staff, volunteers, and money). As some of the discussed recommendations may be challenging for some shelters to implement, it is encouraged for shelters to follow the recommendations that best fit the resource availability of their shelter (5). Some easy and cost-effective solutions that can be readily implemented include using an indirect, non-threatening approach, provisioning of treats, performing the exam on a non-slip sanitizable surface (e.g., yoga mat), and using the least amount of restraint necessary during handling, with the prioritization of staff training on behavior and welfare.

Several limitations may have influenced study findings. Results from this survey are not representative of the entirety of Texas, nor can it be generalized to all shelters within the United States. This is the first study to explore intake procedures in animal shelters; therefore, despite the small sample, data obtained allows us to gain insight into how Texas shelters conduct intake exams and can be used to support future analytical research and targeted intervention strategies to improve the intake experience and support shelter animal welfare. Furthermore, our survey may be susceptible to non-response bias as outreach to shelters located in small towns was challenging, thus it is possible there is an overrepresentation of shelters within more populated cities with more resources. It is also possible that those who believe they use best practice intake policies were more likely to participate. To reduce selection bias as much as possible, we offered a monetary gift card incentive to support their shelter’s needs. The survey respondents may have also answered questions more favorably because of social desirability bias; however, this was mitigated through assuring anonymity.

The aim of this research was to explore the current intake practices used by Texas animal shelters, to identify possible approaches to mitigate dog stress during intake. Techniques employed during intake align with current recommendations to mitigate canine stress. However, results also highlight the need for increased awareness and continued education for shelter staff regarding the benefits of using low-stress handling techniques when dogs are fearful or aggressive and on animal behavior and welfare. Future research is needed to provide evidence-based recommendation on strategies to reduce dog stress during intake, followed by effective knowledge transfer on practical and low-cost ways to implement optimal handling and stress-reducing strategies in shelter settings.

## Data availability statement

The raw data supporting the conclusions of this article will be made available by the authors, without undue reservation.

## Ethics statement

The studies involving humans were approved by Institutional Review Boards (IRB# 2021-844) at Texas Tech University in Lubbock, Texas, United States. The studies were conducted in accordance with the local legislation and institutional requirements. The participants provided their written informed consent to participate in this study.

## Author contributions

MC: Writing – original draft, Writing – review & editing. AB: Data curation, Investigation, Methodology, Writing – original draft, Writing – review & editing. AC: Methodology, Writing – review & editing. ADC: Methodology, Writing – review & editing. HP: Methodology, Writing – review & editing. AS: Conceptualization, Data curation, Funding acquisition, Investigation, Methodology, Project administration, Supervision, Writing – original draft, Writing – review & editing.
