# Supplementary material for: Cross-sectional survey exploring current intake practices for dogs admitted to animal shelters in Texas: a descriptive study
Source: Front Vet Sci. 2023 Dec 19;10:1296425. doi: 10.3389/fvets.2023.1296425 (PMC10763234; doi:10.3389/fvets.2023.1296425)
Supplement: Supplementary file 1 [file Data_Sheet_1.docx]

S1: Survey: Exploring Shelter Intake Procedures

1. Are you 18 years of age or older?

- Yes
- No

2. Are you responsible for completing the intake process for dogs at an animal shelter?

- Yes
- No

3. What county is your shelter located in? [Dropdown of all Texas counties]

4. What type of shelter do you work for?

- Municipal
- Private
- Other __________________________________________________

5. What is the specific type of shelter you work for?

- SPCA
- Humane Society
- Other __________________________________________________

6. Are the personnel responsible for intake required to complete continued education related to dog behavior and welfare?

- Yes
- No
- Unsure
- Not encouraged/no opportunity
- Not applicable

**Part I: Intake procedures and Information collected**

7. Are owner surrenders scheduled?

- Yes, with the exception of emergencies
- No, never
- Unsure

8. For owner surrender cases, which of the following **general** information is collected from the dog owner? (Select all that apply)

- Dog name
- Dog age
- Breed
- Dog sex
- Duration of ownership
- Reason for surrender
- Bite history
- Number of daily walks
- Duration of time left alone on a daily basis
- If they are housetrained
- If they are crate trained
- Type of food provided (e.g., raw diet, kibble, wet only, mixed)
- Other, please specify:________________________________________________

9. For owner surrender cases, which of the following **household** information is collected from the dog owner? (Select all that apply)

- Duration of ownership
- Number of adults (18+) in the home
- Number of children (under 18) in the home
- Dog's behavior towards people in the home
- Dog's behavior towards children in the home
- Number of other pets they lived with
- Type of animals they lived with (e.g., other dogs, cats, birds, rabbits)
- Dog's behavior towards other animals in the home
- Type of dwelling (e.g., apartment, semi-detached, detached)
- Where the dog sleeps at night
- Where the dog stays when home alone
- Where the dog primarily lives (e.g., inside, tethered in a shelter outdoors, tethered without a shelter outdoors)
- Other, please specify: _______________________________________________

10. For owner surrender cases, are owners asked if there is something that would help them keep their dog?

- Yes, always
- Sometimes, it depends on the following factors: _________________________________
- No, never
- Unsure

11. After the animal is surrendered, is there any follow up with previous owners?

- Yes, always
- Sometimes, it depends on the following factors: ________________________________
- No, never
- Unsure

12. Please select the medical history information that is collected during intake. (Select all that apply)

- Pre-existing conditions
- Vaccine record
- Neuter status
- Known allergies
- Cosmetic surgeries (e.g., ear cropping, tail docking)
- Medical history is not collected
- Other, please specify: _______________________________________________

13. For owner surrender cases, which of the following information is collected from the current owner related to the dog's **health** status? (Select all that apply)

- Gastrointestinal issues (e.g. diarrhea, vomiting, swallowed a foreign object such as a sock or poison)
- Musculoskeletal issues (e.g. hip dysplasia, ruptured cruciate ligament, lameness, broken bone)
- Skin condition (e.g. allergies, dermatitis, ear infection, fleas)
- Metabolic/Endocrine issues (e.g. diabetes, hypothyroidism, pancreatitis)
- Respiratory issues (e.g. kennel cough, pneumonia)
- Cardiovascular issues (e.g. heart disease, heart worm)
- Neurological issues (e.g. epilepsy, wobblers)
- Condition causing acute pain
- Condition causing chronic pain
- Impaired vision
- Impaired hearing
- Other, please specify: __________________________________________________
- No health information is collected

14. If the reason for surrendering is **health** related, are interventions in place to assist the current owner in managing their dog's health issues?

- Yes, always
- Sometimes, it depends on the following factors: _______________________________
- No, never
- Unsure

15. For owner surrender cases, which of the following information is collected from the current owner related to the dog's **behavior**? (Select all that apply)

- Fearful tendencies
- Stranger-directed aggression
- Owner-directed aggression
- Dog-directed aggression
- Resource guarding (e.g., protective of toys)
- Separation anxiety (e.g., vocalizes/destructive when left alone)
- Handling sensitivity (e.g., sensitive to hind legs touched)
- Housesoiling issues (inappropriate elimination in the home)
- Excessive vocalization
- Destructiveness (e.g., destroys toys, household belongings)
- Noise phobic (e.g., fearful of thunderstorms)
- Chasing behavior (e.g., prey drive)
- Escape artist
- Other, please specify: __________________________________________________
- No behavior information is collected

16. If the reason for surrendering is **behavior** related, are interventions in place to assist the current owner in managing their dog's behavioral issues?

- Yes, always
- Sometimes, it depends on the following factors: ________________________________
- No, never
- Unsure

17. Are in-shelter behavior assessments conducted at any point while the dog is in the shelter?

- Yes, always
- Sometimes, it depends on the following factors: _____________________________
- No, never
- Unsure

**Part II: Intake Exam**
 
Thinking back to all intake exams, please respond to the following questions regarding an 'average' intake exam.

18. Are there designated rooms for intake exams?

- Yes
- No

19. Does any part of the intake exam occur outside the facility?

- Yes, always
- Sometimes, it depends on the following factors: _____________________________
- No, never
- Unsure

20. If portions of the exam occur outside, please explain the circumstances under which this would occur.

________________________________________________________________

21. Who completes the intake exams?

- Veterinarian
- Veterinary technician
- Animal control officer
- Animal care attendant/worker
- Other, please specify: __________________________________________________

22. Which of the following vaccines are administered?

- DHPP (Distemper, Hepatitis, Parainfluenza, Parvovirus)
- Bordetella
- Rabies
- Canine influenza vaccine
- None
- Other, please specify: __________________________________________________

23. Do you detect for the presence of the following conditions during the intake exam? (Select all that apply)

- Musculoskeletal issues (e.g. hip dysplasia, lameness, broken bone)
- Respiratory issues (e.g. pneumonia)
- Cardiovascular issues (e.g. heart irregularities)
- Neurological issues (e.g. head tilt)
- Intestinal issues (e.g., hookworm, giardia)
- Skin condition (e.g. fungal infection, fleas, mange)
- Heartworm
- Parvovirus
- Underweight
- Fever
- Other, please specify: __________________________________________________
- No conditions are assessed

24. Is flea/tick prevention or treatment provided during the intake exam?

- Yes, always
- Sometimes, it depends on the following factors: _____________________________
- No, never

25. Is any deworming agent provided during the intake exam?

- Yes, always
- Sometimes, it depends on the following factors: __________________________________________________
- No, never

26. If no microchip is detected, are dogs microchipped during the intake exam?

- Yes
- No

27. During the intake exam, which of the following techniques are applied? (Select all that apply)

- Dogs are allowed to explore the examination room for the first few minutes before the examination begins
- Dogs are given lots of attention (e.g. treats, petting, soothing voice)
- Dogs are directly approached by the examiner (standing/walking directly towards the dog)
- Dogs are indirectly approached by the examiner (crouching/kneeling on the ground)
- Other, please specify: __________________________________________________

28. On average, how many personnel are present during the intake exam?

- 1
- 2-3
- >3

29. For intake exams for **large** dogs, please select the most appropriate location of the exam.

- On table with traction surface
- On table without traction surface
- On the ground, with leash tethered to wall
- On the ground, untethered

30. For intake exams for **small** dogs, please select the most appropriate location of the exam.

- On table with traction surface
- On table without traction surface
- On the ground, with leash tethered to wall
- On the ground, untethered

31. Which tools are applied when/if the dog is **calm / fearful / aggressive** towards the shelter staff during the intake exam?  (Select all that apply)

**Calm:** relaxed, no signs of aggression or fear-related behaviors

**Fear:** lowered posture, ears back, tail tucked, whimpering or whining, shaking or trembling, attempts to hide or escape

**Aggression:** fear-related behaviors plus baring teeth, attempting to bite, growling, lunging

|  | Calm | Fearful | Aggressive |
| --- | --- | --- | --- |
| Muzzle |  |  |  |
| Chemical restraint (e.g. sedation) |  |  |  |
| Dog appeasing pheromones (e.g. adaptil) |  |  |  |
| Food and attention (e.g. treats, petting) |  |  |  |
| Additional handlers requested |  |  |  |
| Additional handlers removed |  |  |  |
| Exam length shortened |  |  |  |
| Calming Cap (soft fabric that covers the dog's eyes) |  |  |  |
| Elizabethan collar (big collar around the dog's head) |  |  |  |
| Catch pole/rabies pole |  |  |  |
| Towel restraint |  |  |  |
| Performing exam on someone's lap |  |  |  |
| Head collar |  |  |  |
| Other, please specify: |  |  |  |

32. If a dog enters the shelter with a known history of aggression (based on intake questionnaire administered), which of the following steps would be taken?

- Additional personnel requested to assist with handling
- A sedative is administered
- Dog is muzzled
- Dog is placed on a catch/rabies pole
- Behavior is briefly assessed by personnel
- Nothing different is performed, each dog is approached the same
- Other, please specify: __________________________________________________

**Part III: Environment**

33. Is there a separate entrance that is used for intake?

- For stray only
- For owner surrender only
- For all intake animals
- No separate entrance

34. Please select where dogs are housed, on average, after completion of intake procedures?

- Isolated in a closed room
- In a kennel within the intake room
- In a kennel within the regular dog room
- Wherever space is available
- Other, please specify: __________________________________________________

Thank you for participating! If you have any comments about the survey, please let us know in the comment box below.

________________________________________________________________

________________________________________________________________

________________________________________________________________
